# Folic acid deficiency exacerbates the inflammatory response of astrocytes after ischemia‐reperfusion by enhancing the interaction between IL‐6 and JAK‐1/pSTAT3

**DOI:** 10.1111/cns.14116

**Published:** 2023-02-16

**Authors:** Man Cheng, Xiaoshan Liang, Linran Shi, Qiang Zhang, Liwen Zhang, Zhongying Gong, Suhui Luo, Xuan Wang, Xumei Zhang

**Affiliations:** ^1^ Department of Nutrition and Food Science, School of Public Health Tianjin Medical University Tianjin China; ^2^ Tianjin Key Laboratory of Environment, Nutrition and Public Health, Center for International Collaborative Research on Environment, Nutrition and Public Health Tianjin Medical University Tianjin China; ^3^ Department of Occupational and Environmental Health School of Public Health, Tianjin Medical University Tianjin China; ^4^ Department of Neurology Tianjin First Center Hospital, School of Medicine, Nankai University Tianjin China

**Keywords:** astrocyte, folic acid deficiency, IL‐6, inflammation, ischemia‐reperfusion injury

## Abstract

**Aim:**

To demonstrate the role of IL‐6 and pSTAT3 in the inflammatory response to cerebral ischemia/reperfusion following folic acid deficiency (FD).

**Methods:**

The middle cerebral artery occlusion/reperfusion (MCAO/R) model was established in adult male Sprague‐Dawley rats in vivo, and cultured primary astrocytes were exposed to oxygen‐glucose deprivation/reoxygenation (OGD/R) to emulate ischemia/reperfusion injury in vitro.

**Results:**

Glial fibrillary acidic protein (GFAP) expression significantly increased in astrocytes of the brain cortex in the MCAO group compared to the SHAM group. Nevertheless, FD did not further promote GFAP expression in astrocytes of rat brain tissue after MCAO. This result was further confirmed in the OGD/R cellular model. In addition, FD did not promote the expressions of TNF‐α and IL‐1β but raised IL‐6 (Peak at 12 h after MCAO) and pSTAT3 (Peak at 24 h after MCAO) levels in the affected cortices of MCAO rats. In the in vitro model, the levels of IL‐6 and pSTAT3 in astrocytes were significantly reduced by treatment with Filgotinib (JAK‐1 inhibitor) but not AG490 (JAK‐2 inhibitor). Moreover, the suppression of IL‐6 expression reduced FD‐induced increases in pSTAT3 and pJAK‐1. In turn, inhibited pSTAT3 expression also depressed the FD‐mediated increase in IL‐6 expression.

**Conclusions:**

FD led to the overproduction of IL‐6 and subsequently increased pSTAT3 levels via JAK‐1 but not JAK‐2, which further promoted increased IL‐6 expression, thereby exacerbating the inflammatory response of primary astrocytes.

## Graphical abstract

Folic acid deficiency raises IL‐6 level in astrocytes following ischemic injury. Folic acid deficiency increases pSTAT3 expression via JAK‐1 in OGD/R‐astrocytes. Crosstalk of IL‐6 and pSTAT3 amplifies the neuroinflammatory response.

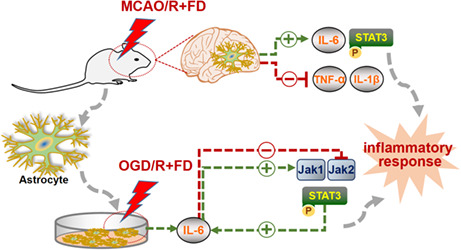

## 1 INTRODUCTION

Cerebral ischemia/reperfusion (I/R), caused by the restoration of blood supply to ischemic brain tissue, is a pathological injury that occurs during the treatment of ischemic stroke and is accompanied by high morbidity and mortality.^1^ There are no specific drugs available to treat I/R injury.^2^ Thus, in such a case, dietary supplements with low side effects may be considered to assist in promoting neurological recovery if supported by substantial scientific evidence.^3^

Folic acid (FA), an essential nutrient in the regular human diet, is strongly associated with neuroinflammation.^4^, ^5^ Research has shown that folic acid deficiency (FD) triggers the activation of the neuroinflammatory cascade in Alzheimer's disease (AD).^6^ In addition, Guest et al. observed a negative correlation between cerebrospinal fluid folate and levels of inflammation within the central nervous system (CNS) in the healthy population.^7^ However, the exact mechanisms underlying the effects of FD on neuroinflammation following cerebral ischemia‐reperfusion have not been fully elucidated. Our previous work suggests that FD may enhance the expression of inflammatory mediators following cerebral hypoxia‐ischemia by activating microglia.^8^ Although astrocytes and microglia are known to be critical regulators of the inflammatory response in the CNS,^9^ the mechanisms by which astrocytes are involved in the effects of FD on stroke recovery require further investigation.

Astrocytes, the most common glial cells in the brain, are key regulators of the inflammatory response in the CNS.^10^ For instance, in the early stages of AD, astrocytes become activated and release interleukins and nitric oxide, exacerbating the neuroinflammatory response.^11^ In experimental autoimmune encephalomyelitis mice, astrocytes produce lactosylceramide, which promotes transcriptional levels of pro‐inflammatory factors such as IL‐1β and nitric oxide synthase in an autocrine manner.^12^ Additionally, astrocyte proliferation is an important pathological feature of stroke. Reactive astrocytes can release pro‐inflammatory cytokines in response to acute ischemia, especially IL‐6, thereby triggering the production of secondary mediators, which may lead to persistent and neurotoxic effects.^13^ Given that FD induces neuroinflammation in CNS disorders, FD may promote inflammatory responses in astrocytes following ischemia‐reperfusion.

Interleukin‐6 (IL‐6)/signal transduction and transcription activator of 3 (STAT3) is an essential intracellular pathway that mediates inflammatory signaling and is a vital signaling component in reactive astrocytes.^14^ As a core upstream regulator of the inflammatory response, IL‐6 promotes inflammatory response waterfalls and simultaneously activates STAT3 via Janus kinases (JAKs). Subsequently, aberrant activation of STAT3 promotes transcriptions and expressions of many genes encoding pro‐inflammatory mediators.^15^ Here, we hypothesize that FD may exacerbate astrocyte injury through IL‐6/pSTAT3 interactions.

In this present study, both the rat middle cerebral artery occlusion/reperfusion (MCAO/R) model and oxygen‐glucose deprivation/reoxygenation (OGD/R)‐treated primary astrocytes were used to observe FD's effects on astrocytes and further explore the underlying molecular mechanisms. The study shows for the first time that FD triggers an inflammatory response in astrocytes after ischemia‐reperfusion through the IL‐6/JAK‐1/pSTAT3 pathway and exacerbates inflammation through the interaction between IL‐6 and pSTAT3. This work will provide new insights into how FD leads to astrocyte injury after ischemic stroke.

## 2 MATERIALS AND METHODS

### 2.1 Animals

SPF male Sprague‐Dawley rats (weighing 160–180 g) were purchased from Peking Wei Tong Lihua Experimental Animal Technology Center (Beijing, China). All animal experiments described in this study were conducted by the Guide for the Care and Use of Laboratory Animals published by the National Institutes of Health (NIH publication no. 80‐23, revised 1996). The experimental animals were randomly divided into five groups: (1) sham‐operated control group (SHAM, *n* = 10), (2) MCAO 12 h group (MCAO 12 h, *n* = 10), (3) MCAO 24 h group (MCAO 24 h, *n* = 10), (4) MCAO 12 h plus folic acid deficient diet group (MCAO 12 h + FD, *n* = 10), (5) MCAO 24 h plus folic acid deficient diet group (MCAO 24 h + FD, *n* = 10). The rats were pretreated with the standard (2 mg/kg) or folic acid deficient diets (<0.2 mg/kg) (Beijing Keao Xieli Feed Co., Ltd.) for 28 days prior to animal operation. All animal protocols were approved by the Animal Ethical and Welfare Committee of Tianjin Nankai Hospital.

### 2.2 Surgical procedures

The MCAO rats were induced by the intraluminal filament technique, as described previously.^16^ After 1 h of MCAO‐induced focal cerebral ischemia, the line was carefully withdrawn to establish reperfusion. The rats were then allowed to recover from anesthesia at 37°C and were sacrificed at 12 h and 24 h after reperfusion for the following experiments.

### 2.3 Cell culture and treatment

Brain tissue was isolated from neonatal Sprague‐Dawley rats (within 24 h) with careful removal of the meninges and subsequently washed three times in Dulbecco's modified eagle's medium (DMEM; Sigma, St. Louis, MO, USA). It was cut into small pieces and then dissociated by incubation with 0.25% parenzyme and 0.02% EDTA for 15 min. After centrifugation and resuspension, the mixed glial cell cultures were kept in T75 culture flasks and fed with DMEM supplemented with 10% fetal bovine serum (FBS). The media were changed twice a week. When cells reached ∼95% confluence, microglia and astrocytes were separated by gentle shaking for 16 h at room temperature. By immunocytochemistry, 95% of cells are GFAP positive. Then, the cells were seeded into the culture flask with normal DMEM (4 mg/L folic acid; Sigma). The model of folic acid deficient primary astrocytes was established through folic acid deficient Dulbecco's modified eagle's medium (0 mg folic acid; Sigma) supplemented with 10% FBS. LMT‐28 (IL‐6 inhibitor, 30 μM),^17^ C188‐9 (STAT3 inhibitor, 30 μM),^18^ Filgotinib (a specific JAK‐1 inhibitor, 10 nM), and AG490 (a specific JAK‐2 inhibitor, 50 μM)^19^, ^20^, ^21^ were used to inhibit the expression of the corresponding proteins.

To imitate the cerebral I/R model in vivo, the cells were induced by OGD/R. The normal medium (containing 10% FBS and 4.5 g/L glucose) was replaced by glucose‐free DMEM (Gibco). Then, the cells were exposed to a three‐gas incubator at 37°C containing 1.0% O_2_ to initiate hypoxia for 1 h, followed by 3 h re‐oxygenation in a normoxia incubator. Normal control cells were incubated in a regular cell culture incubator under normoxic conditions.

### 2.4 Immunofluorescence

Immunofluorescence staining of the rat brain sections was performed as previously described.^22^ In brief, the sections were de‐waxed and hydrated to dispose of 3% H_2_O_2_ for 10 min at room temperature, repaired by citric acid antigen, and blocked with goat serum for 1 h at 37°C. Then, they were incubated overnight 4°C with the primary antibodies (mouse anti‐IL‐6, rabbit anti‐TNF‐α, rabbit anti‐IL‐1β, rabbit anti‐GFAP, 1:200, Abcam; mouse anti‐GFAP, rabbit anti‐pSTAT3, Cell Signaling Technology). The next day, the sections were washed in PBS and then incubated with the secondary antibodies (1:100, Zhongshan Gold bridge Biotechnology, China) for 1 h at room temperature. Then, they were mounted with DAPI (4′,6‐diamidino‐2‐phenylindole) and ProLong Gold™ Antifade Reagent (catalog number P36931; Life Technologies, Carlsbad, CA) and subsequently examined in a fluorescence microscope (IX81; Olympus). The positive cells were counted by Image Pro Plus 6.0.

### 2.5 Western blot

Western blot was performed as previously described.^22^ Total proteins (20 μg) were subjected to SDS‐PAGE and then transferred onto a polyvinyl indene difluoride membrane (PVDF; Millipore, Billerica, MA, USA). Nonspecific binding was blocked with PBST (0.5% Tween 20 in PBS) containing 5% non‐fat milk (Shandong Sparkjade Biotechnology Co., Ltd.) for 1 h at room temperature. The membranes were then incubated overnight at 4°C with individual primary antibodies in PBST containing 1% non‐fat milk (mouse anti‐IL‐6, rabbit anti‐TNF‐α, rabbit anti‐IL‐1β, rabbit anti‐GFAP, 1:1000, Abcam; mouse anti‐GFAP, rabbit anti‐pSTAT3, mouse anti‐STAT3, rabbit anti‐JAK‐1, rabbit anti‐JAK‐2, 1:1000, Cell Signaling Technology, rabbit anti‐β‐actin, Biosynthesis Biotechnology Inc., Beijing, China). Following three washes with PBST, the membranes were then incubated with the secondary antibodies (HRP‐linked anti‐rabbit IgG; HRP‐linked anti‐mouse IgG; 1:2000; Cell Signaling Technology). Then, the proteins were detected by chemiluminescence reagents (Millipore) and observed using a ChemiDoc™ XRS+ Imaging System (Bio‐RAD, Hercules, USA). The protein levels were quantified by densitometry using Image J 1.4.3.67.

### 2.6 Statistical analysis

SPSS V.20 and GraphPad Prism V.9.0 were used for the statistical analysis. All quantitative data were expressed as mean ± standard deviation ($\bar{x}$±s). All data were tested for normality using the Shapiro‐Wilk test. One‐way ANOVA was used to assess the statistical significance of the differences among different experimental groups, followed by Student‐Newman‐Keuls multiple‐range tests. *p* < 0.05 was assumed statistically significant.

## 3 RESULTS

### 3.1 Folic acid deficiency does not further promote GFAP expression raised by ischemic injury in vitro and in vivo

**FIGURE 1:**
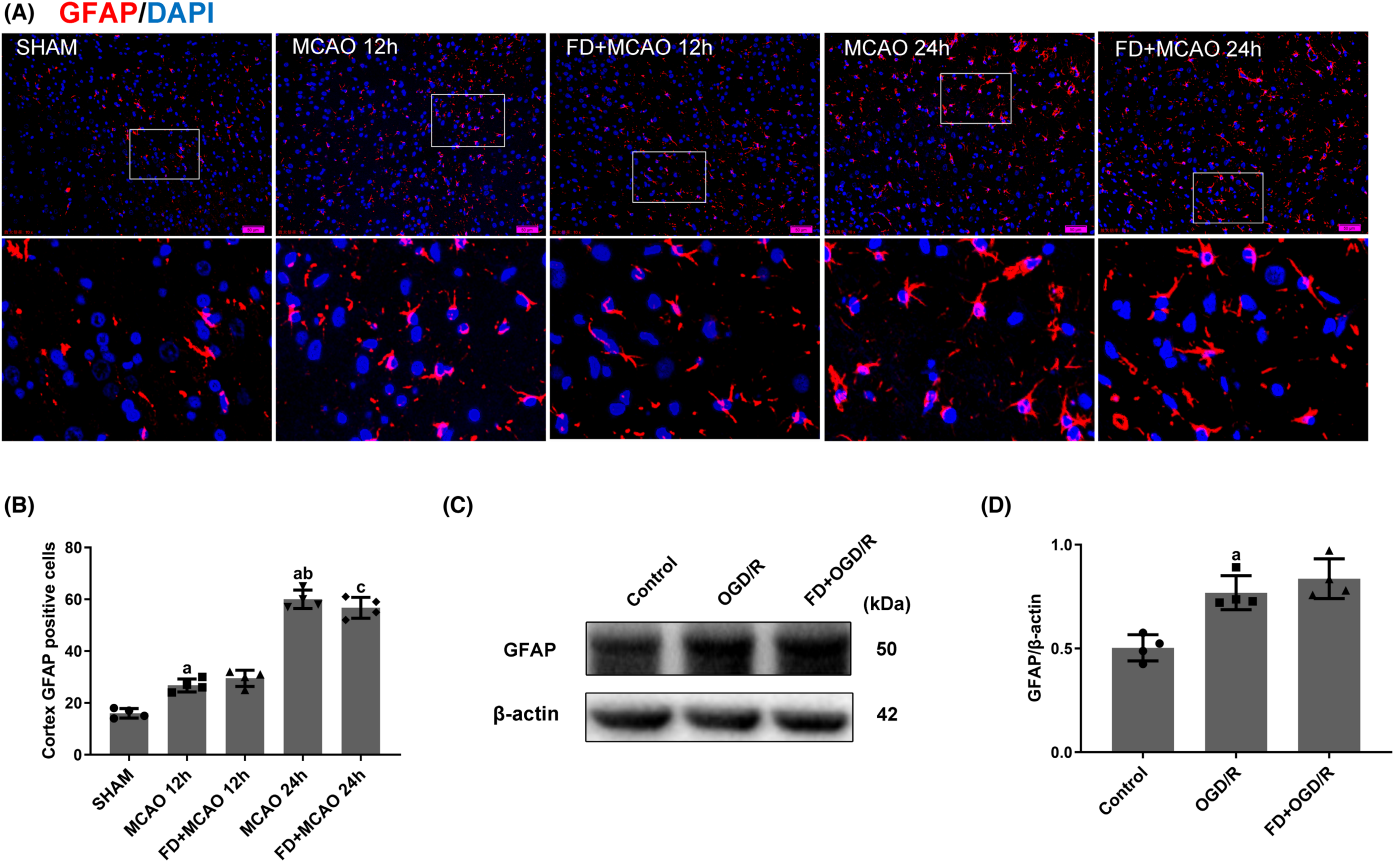
Effect of folic acid deficiency on GFAP expression in astrocytes. (A, B) Double labeling immunofluorescence of DAPI (blue) and GFAP (red) in SHAM, MCAO, and MCAO+FD group rats after 12 h and 24 h of reperfusion. Data shown are mean ± SEM (*n* = 4). ^a^ *p* <0.05: Compared to SHAM, ^b^ *p* <0.05: Compared to MCAO 12 h, ^c^ *p* <0.05: Compared to MCAO+FD 12 h. The cells were harvested after incubating with normal DMEM, normal DMEM and OGD/R, folic acid deficient DMEM and OGD/R. (C, D) Western blot analyses of GFAP and β‐Actin. Data shown are mean ± SEM (*n* = 4). ^a^ *p* <0.05: Compared to Control.

Several lines of evidence support that in response to stroke, astrocytes convert to a reactive phenotype chiefly characterized by up‐regulation of GFAP and cellular hypertrophy.^23^ To determine the effect of FD on the reactive astrocytes, GFAP protein expression was detected in the MCAO rat brain and cultured primary astrocytes by immunohistochemical staining and western blot. The results showed an evident increase of GFAP expression at 12 h of reperfusion compared to the SHAM group, and further increased by 24 h (*p* < 0.05; Figure 1A, B). This result was further confirmed in in vitro OGD/R cellular model (*p* < 0.05; Figure 1C, D). However, FD did not significantly alter GFAP expression compared to the MCAO/R (or OGD/R) group.

### 3.2 Folic acid deficiency promotes IL‐6 but not TNF‐α and IL‐1β expressions in astrocytes following ischemic injury

**FIGURE 2:**
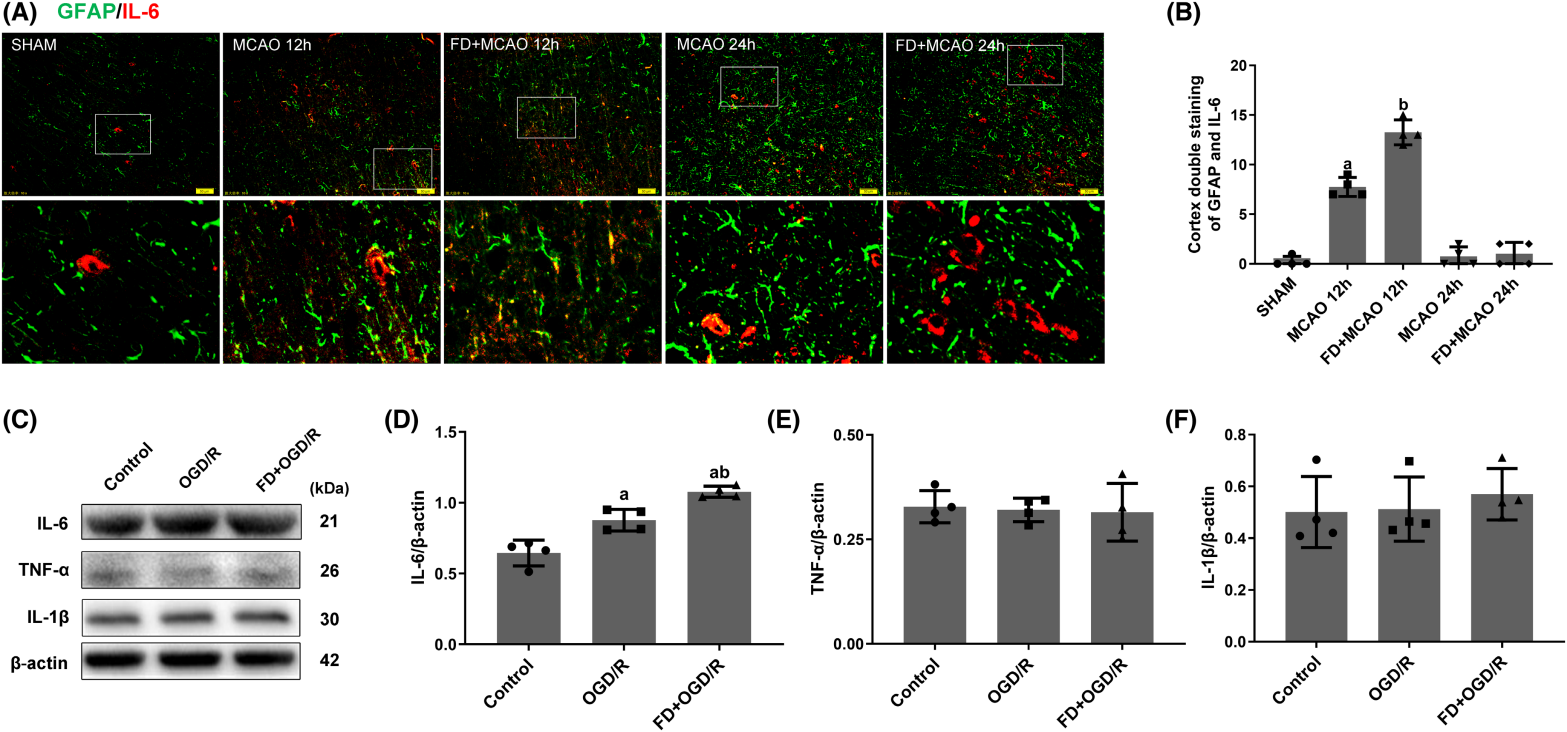
Effect of folic acid deficiency on IL‐6, IL‐1β and TNF‐α expressions in astrocytes. (A, B) Double labeling immunofluorescence of IL‐6 (red) and GFAP (green) in SHAM, MCAO, and MCAO+FD group rats after 12 h and 24 h of reperfusion. Data shown are mean ± SEM (*n* = 4). ^a^ *p* <0.05: Compared to SHAM, ^b^ *p* <0.05: Compared to MCAO 12 h. (C) Western blot analyses of IL‐6, IL‐1β, TNF‐α and β‐Actin. (D‐F) Bar chart showing the IL‐6/β‐Actin, TNF‐α/β‐Actin and IL‐1/β‐Actin ratio from the western blot analysis. Data shown are mean ± SEM (*n* = 4). ^a^ *p* <0.05 compared to Control. ^b^ *p* <0.05: Compared to OGD/R group.

Astrocyte‐derived neuroinflammation has been identified as a potential contributor to brain injury.^24^ To determine whether FD could modulate astrocyte‐mediated neuroinflammation, three pro‐inflammatory cytokines, TNF‐α, IL‐1β, and IL‐6, were detected by immunofluorescence double‐labeling and western blot analysis. As shown in Figure 2, the number of GFAP/IL‐6‐positive cells were significantly increased in the MCAO 12 h group compared to the SHAM group (*p* < 0.05) but reduced to almost the same level as in the SHAM group after 24 h of reperfusion. The number of GFAP/IL‐6‐positive cells was further raised by FD intervention compared to the MCAO group (*p* < 0.05). In line with what was observed in vivo, FD promoted IL‐6, but not TNF‐α or IL‐1β levels in primary astrocytes exposed to OGD/R compared to the OGD/R alone (*p* < 0.05; Figure 2C, D).

### 3.3 Folic acid deficiency results in an increase in pSTAT3 expression in the astrocytes following ischemic injury

**FIGURE 3:**
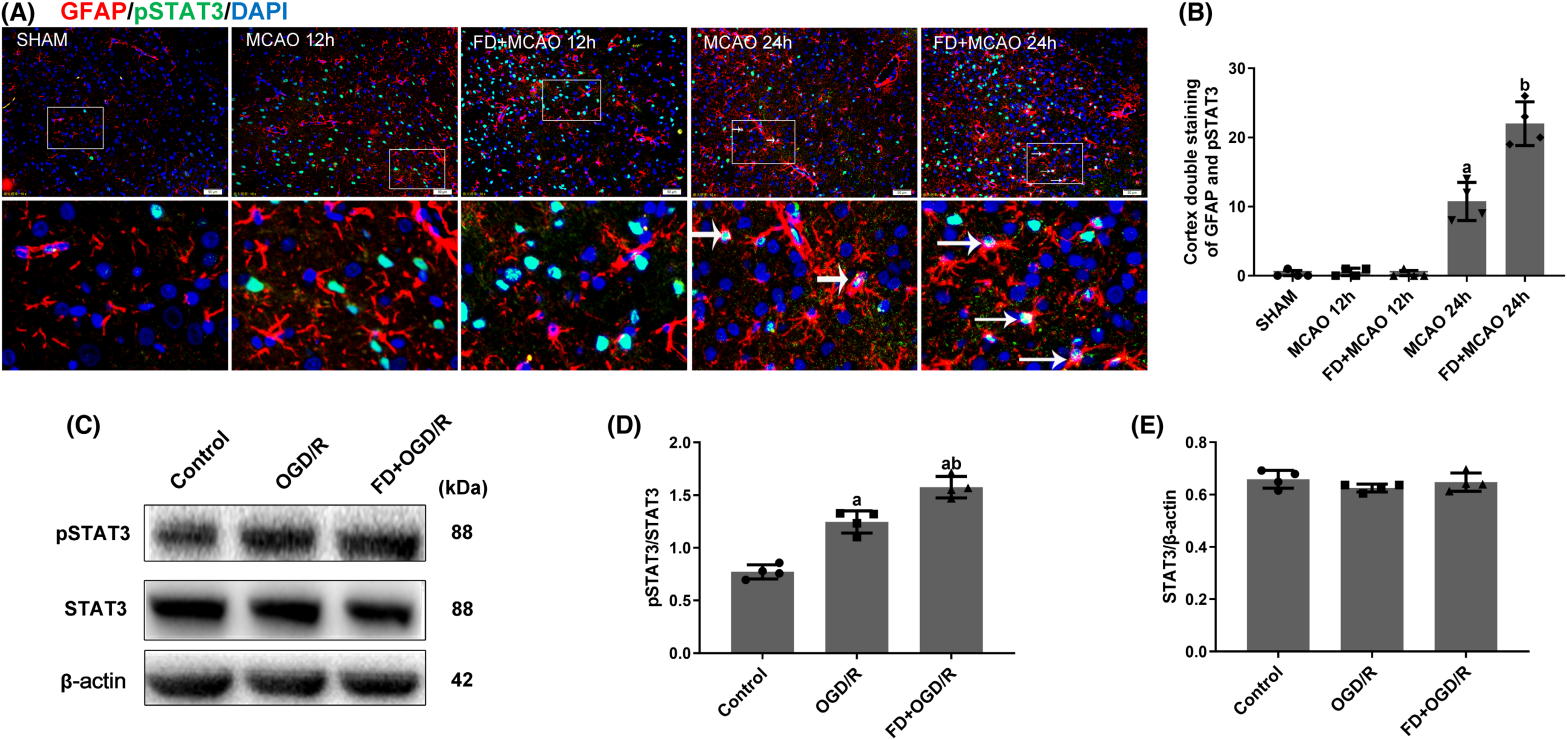
Effect of folic acid deficiency on p‐STAT3 expression in astrocytes. (A, B) Double labeling immunofluorescence of DAPI (blue), GFAP (red) and p‐STAT3 (green) in SHAM, MCAO, and MCAO+FD group rats after12 h and 24 h of reperfusion. Data are shown as mean ± SEM (*n* = 4). ^a^ *p* <0.05: Compared to SHAM, ^b^ *p* <0.05: Compared to MCAO 24 h. (C) Western blot analyses of pSTAT3, STAT3, and β‐Actin. (D) Representative ratios of pSTAT3 to STAT3. (E) Representative ratios of STAT3 to β‐Actin. Data are shown as mean ± SEM (*n* = 4). ^a^ *p* <0.05: Compared to Control. ^b^ *p* <0.05: Compared to OGD/R group.

Accumulated evidence suggested that activation of STAT3 plays an important role in IL‐6‐mediated inflammation.^25^ The effect of FD on pSTAT3 expression in astrocytes was examined. The results indicated that pSTAT3 expression did not change significantly at 12 h after reperfusion but increased significantly after 24 h reperfusion compared to the SHAM group. FD further increased the number of GFAP/pSTAT3 double‐positive cells in the ischemic brain compared with the MCAO/R group. Similarly, FD promoted pSTAT3 expression raised by OGD/R in primary astrocytes (*p* < 0.05; Figure 3 D‐E).

### 3.4 Folic acid deficiency increases the level of pSTAT3 through JAK‐1 but not JAK‐2

**FIGURE 4:**
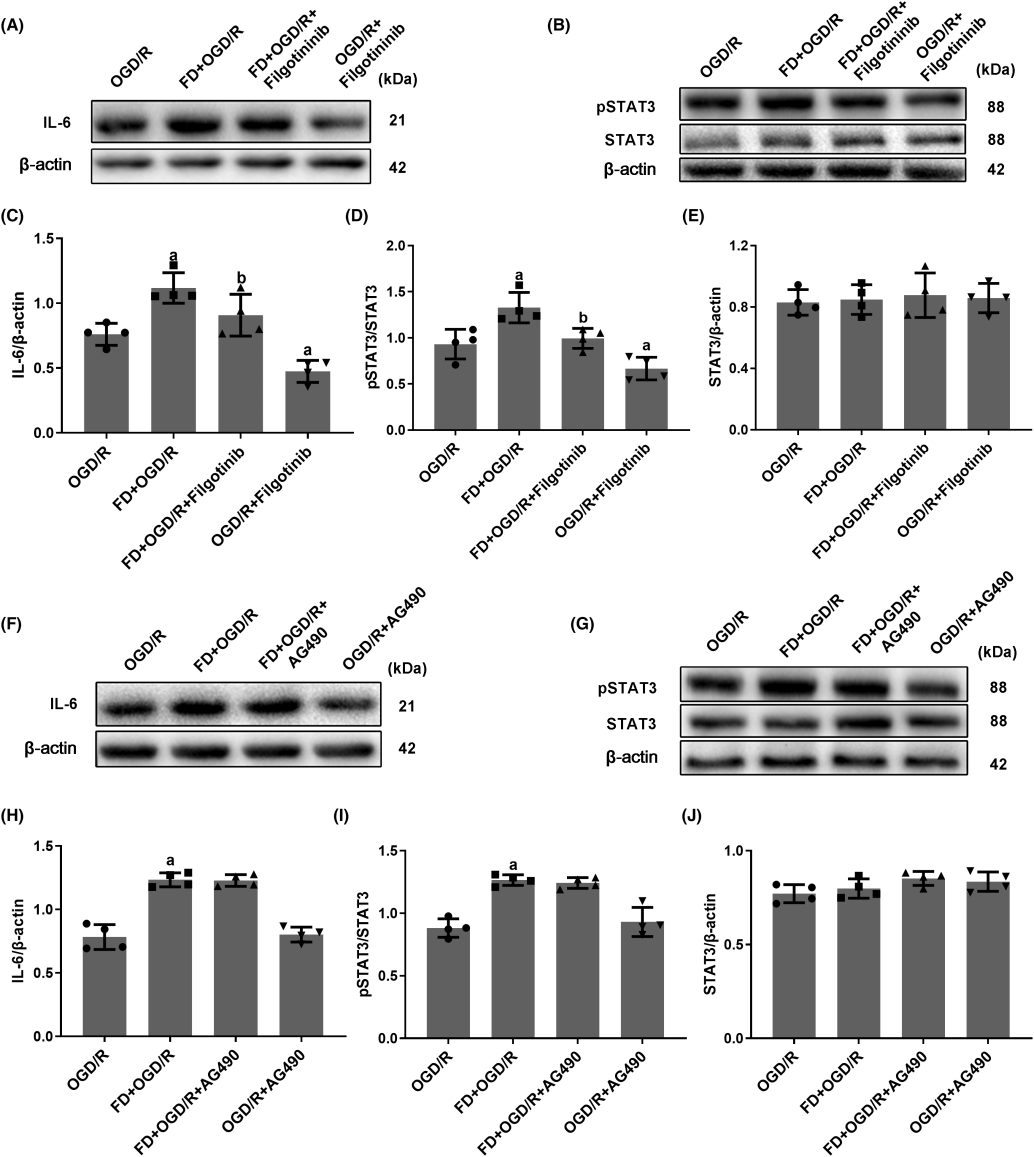
Folic acid deficiency regulates the expression of pSTAT3 in primary astrocytes exposed to hypoxia and glucose deficiency via the JAK‐1 pathway. (A‐E) The cells were harvested after incubating with Filgotinib (JAK‐1 inhibitor). The protein expressions of IL‐6 (A), pSTAT3, and STAT3 (B) were detected by western blot. Bar graphs show the relative levels of IL‐6 (normalized to β‐Actin) (C), pSTAT3 (normalized to STAT3) (D), and STAT3 (normalized to β‐Actin) (E). (F‐J) The cells were harvested after incubating with AG490 (JAK‐2 inhibitor). The protein expressions of IL‐6 (F), pSTAT3 and STAT3 (G) were detected by western blot. Bar graphs show the relative level of IL‐6 (normalized to β‐Actin) (H), pSTAT3 (normalized to STAT3) (I) and STAT3 (normalized to β‐Actin) (J). Data are shown as mean ± SEM (*n* = 4). ^a^ *p* <0.05: Compared to OGD/R group. ^b^ *p* <0.05: Compared to FD + OGD/R group.

In inflammatory diseases, STAT3 is usually activated by phosphorylation through the activation of non‐receptor protein tyrosine kinases JAKs.^15^ To elucidate whether FD upregulated pSTAT3 expression in a JAK‐dependent manner, the expression of pSTAT3 was detected. As shown in Figure 4, Filgotinib administration significantly reduced the levels of IL‐6 and pSTAT3, but AG490 treatment did not reveal any significant changes in the expression of IL‐6 or pSTAT3. Our results proved that FD increased the level of pSTAT3 through JAK‐1 instead of JAK‐2.

### 3.5 Interaction between IL‐6 and pSTAT3 in hypoxic and glucose‐deficient astrocytes after folic acid deficiency

**FIGURE 5:**
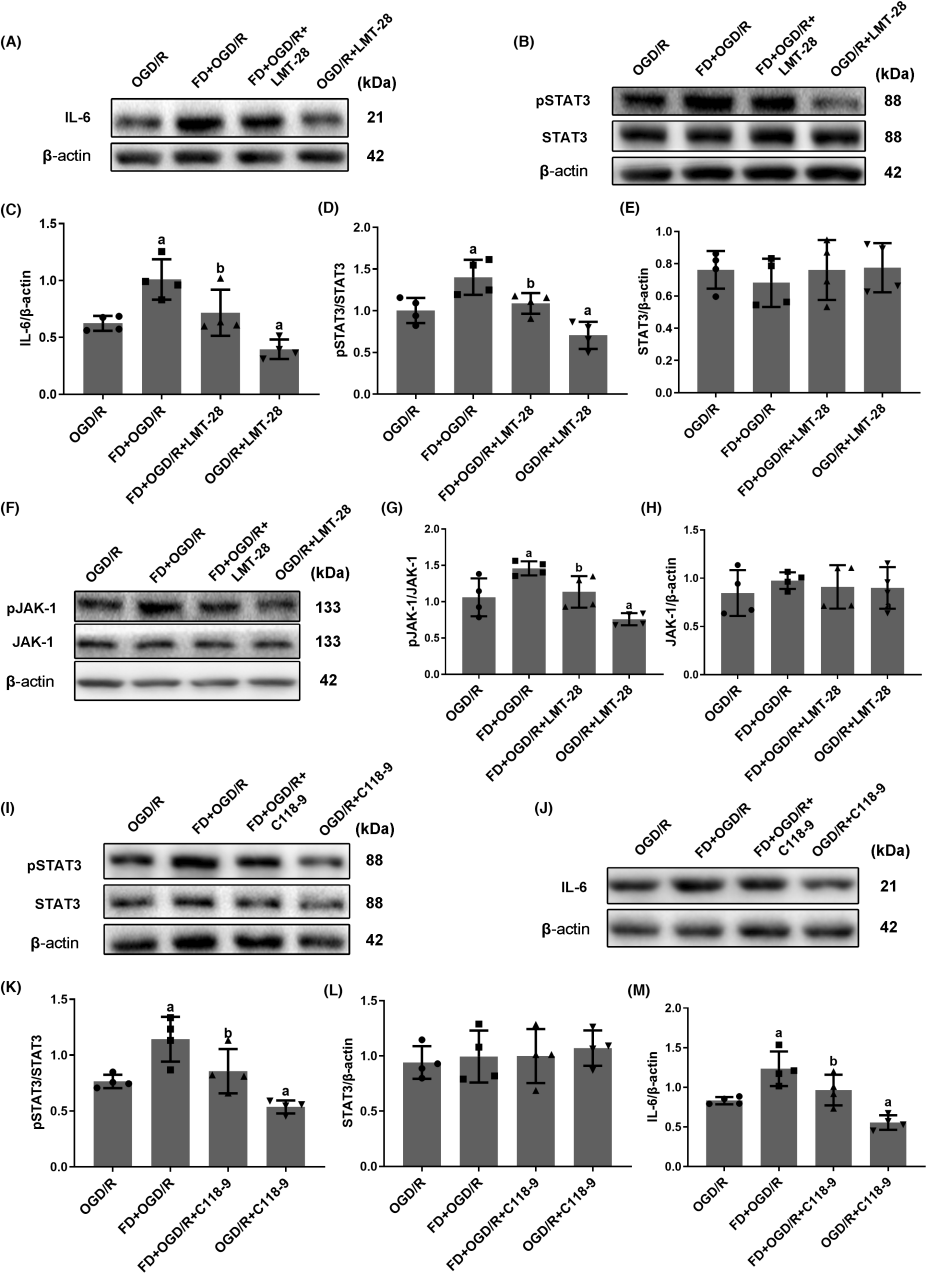
The potential association of IL‐6 and pSTAT3 in hypoxic and glucose‐deficient astrocytes after FD intervention. (A‐H) The cells were harvested after incubating with LMT‐28 (IL‐6 inhibitor). The protein expressions of IL‐6 (A), pSTAT3, STAT3 (B), pJAK‐1 and JAK‐1 (F) were detected by western blot. Bar graphs show the relative levels of IL‐6 (normalized to β‐Actin) (C), pSTAT3 (normalized to STAT3) (D), STAT3 (normalized to β‐Actin) (E), pJAK‐1 (normalized to JAK‐1) (G) and JAK‐1 (normalized to β‐Actin) (H). (I‐M) The cells were harvested after incubating with C118‐9 (STAT3 inhibitor). The protein expressions of pSTAT3, STAT3 (I), and IL‐6 (J) were detected by western blot. Bar graphs represented the relative levels of pSTAT3 (normalized to STAT3) (K), STAT3 (normalized to β‐Actin) (L) and IL‐6 (normalized to β‐Actin) (M). Data shown are mean ± SEM (*n* = 4). ^a^ *p* <0.05: Compared to OGD/R group, ^b^ *p* <0.05: Compared to FD + OGD/R group.

STAT3, a key transcription factor, is involved in mediating acute inflammatory response activities located downstream of IL‐6.^25^ To explore the potential correlation between IL‐6 and pJAK‐1/pSTAT3, the cells were first treated with LMT‐28. The Western blot results in Figure 5 showed that treatment with IL‐6 inhibitor significantly inhibited both pSTAT3 and pJAK‐1 expressions after OGD/R treatment in astrocytes (*p* < 0.05; Figure 5A‐H). Then, whether pSTAT3 affected IL‐6 expression was assessed by adding C188‐9 to OGD/R‐treated astrocytes. As shown in Figure 5 I‐M, the expression of IL‐6 was also inhibited after adding STAT3 inhibitor (*p* < 0.05). Briefly, the results showed that inhibiting IL‐6 expression reduces pSTAT3 levels, while pSTAT3 inhibition also decreases IL‐6 expression, suggesting a positive feedback loop between these factors.

## 4 DISCUSSION

Inadequate levels of folic acid are associated with an increased risk of neurodegenerative diseases and cerebrovascular disease.^26^ However, the exact mechanisms still need to be determined. Previous efforts have traditionally focused on the exploration of intrinsic neuronal mechanisms. In recent years, some studies have discovered the critical role of astrocytes in ischemic lesions. For instance, reactive astrocytes produce and release pro‐inflammatory mediators, which may lead to neuronal death and infarct progression.^27^ In the present study, we focused on astrocytes in order to gain insight into novel mechanisms by which FD affects neurological function. This is the first evidence that the IL‐6/JAK‐1/pSTAT3 pathway triggered the inflammatory response of astrocytes in the presence of FD. Notably, FD leads to the overproduction of IL‐6 in the astrocytes, which next activates pSTAT3, leading to more IL‐6 production and release. This interaction between IL‐6 and pSTAT3 may amplify neuroinflammatory responses, leading to secondary brain damage.

There is strong experimental evidence that folic acid affects inflammation in the central nervous system; it also suggests intricate mechanisms by which this occurs. For instance, folic acid reduces hippocampal myeloperoxidase activity to alleviate neuroinflammation and improve memory impairment in sepsis‐induced rats.^28^ Another in vitro study indicated that lipopolysaccharide‐activated microglia respond less inflammatory to folic acid because it inhibits the activation of NF‐kB and JNK and upregulates p38 MAPK phosphorylation.^4^ Besides, our previous work has shown that FD enhanced microglia immune responses via the Notch1/nuclear factor kappa B p65 pathway to increase brain injury.^8^ The current study investigated the effect of FD on the astrocytes under ischemia‐reperfusion. We revealed that FD promoted the inflammatory response of astrocytes by exacerbating the interaction between IL‐6 and JAK‐1/pSTAT3. Multiple signaling molecules may be involved in FD's activation of neuroinflammation, which may vary depending on different cell types or disease conditions.

Both JAK1 and JAK2 have been proven to be associated with the IL‐6 activation of STAT3 pathway.^29^ However, those two Janus kinases are known to each have different roles in different pathological and physiological processes. For instance, Yang et al. demonstrated that the release of IL‐6 activated the JAK2/STAT3 pathway to aggravate neuronal degeneration in mice with Parkinson's disease.^30^ Whereas, increased IL‐6 expression exacerbates the inflammatory response of macrophages through the JAK1/STAT3 pathway in mouse models of ulcerative colitis.^31^ To elucidate the exact pathway by which FD upregulates pSTAT3 expression, we blocked the activation of JAK‐1 and JAK‐2 using Filgotinib and AG490, respectively. The results demonstrate that FD‐induced pSTAT3 expression was significantly inhibited in OGD/R‐treated astrocytes after blocking the activation of JAK‐1 but not JAK‐2. Although different JAKs may have overlapping roles, each has an important role in mediating signaling. It has been shown that JAK1 is a central protein in the inflammatory response cytokine network and can produce pro‐inflammatory activity.^32^ Nevertheless, JAK‐2 is mainly involved in processes such as mitotic reorganization and histone modification and is essential for bone marrow and platelet production.^33^ These support our findings that FD exacerbates the inflammatory response in astrocytes via the IL‐6/JAK‐1/pSTAT3 pathway after ischemia‐reperfusion.

There is a complex regulatory relationship between IL‐6 and pSTAT3. As a transcription factor, STAT3 is involved in mediating the acute inflammatory response to the genes associated downstream of IL‐6.^34^ Binding of IL‐6 to its receptor activates the phosphorylation of STAT3. pSTAT3 then binds to DNA and increases the expression of cytokine genes, resulting in the production of more interleukins. This vicious cycle leads to persistent nervous system inflammation unless effectively controlled.^35^ This is consistent with our results that there may be an interaction between IL‐6 and pSTAT3 expressions in folic acid deficient OGD/R astrocytes and that the malignant feedback between them may play an essential role in FD‐mediated astrocyte injury.

In general, STAT3 is a vital player in the proliferative response of reactive astrocytes.^23^ Also, STAT3 is one of the transcription factors of GFAP and the increase of GFAP expression tends to be accompanied by STAT3 activation.^36^ A noteworthy point to ponder is that FD promoted p‐STAT3 expression but not GFAP activation in our study. This is possible because astrocyte activation is finely regulated by many intracellular and extracellular signaling molecules, such as TGF‐β, NF‐κB, and STAT3.^37^, ^38^, ^39^ However, some regulatory factors, such as the FGF signaling pathway, inhibit the activation of astrocytes.^40^ Therefore, we speculate that, in the case of FD, the activation of some inhibitory factors may be involved and thus FD did not further activate GFAP.

Additionally, Takumi Takizawa et al. proved that abnormal methylation of the STAT3 binding element in the GFAP promoter in astrocytes prevents the binding of STAT3, thereby inhibiting GFAP transcription.^41^ Besides, the AP‐1 transcription factor is essential for promoting the upregulation of GFAP genes in response to injury.^42^ Folic acid is involved in DNA synthesis and methylation and thus plays a crucial role in maintaining genomic stability.^43^ Therefore, in the presence of FD, abnormal synthesis of key transcription factors and abnormal methylation of binding sites may be involved, failing to promote GFAP expression.

Nevertheless, there are two main limitations for consideration. Firstly, the present study focused on the early molecular changes caused by FD at the onset of cerebral infarction. Considering that post‐stroke neuroinflammation is a highly dynamic and complex adaptive process,^44^ long‐term FD intervention may be necessary for further behavioral observation and the exploration of molecular mechanisms at the later stage of disease in the future study. Secondly, both astrocytes and microglia mediate inflammatory responses through related molecules in response to the stress of ischemic brain injury.^45^ Further evidence supported that there are reciprocal interactions between microglia and astrocytes during neuroinflammation.^46^ Our previous and present studies respectively verified that FD exacerbates the inflammatory response of microglia and astrocytes after ischemia‐reperfusion.^8^ However, in the light of the existing experiment data, we are unable to determine whether microglia or astrocytes play a more critical role during the regulation of FD on neuroinflammation, and whether FD affects the interaction between the two types of glials or not.

In conclusion, this study found that in the context of ischemia‐reperfusion, folic acid deficiency may trigger astrocytes' inflammatory response via the IL‐6/JAK‐1/pSTAT3 pathway. Furthermore, the interaction between IL‐6 and pSTAT3 may amplify the neuroinflammatory response, leading to secondary brain injury. Therefore, specific inhibition of the IL‐6/JAK‐1/pSTAT3 pathway in astrocytes is a potential therapeutic approach to alleviate the progression of ischemic stroke caused by folic acid deficiency. This also suggests that folic acid supplementation is a potential preventive and therapeutic strategy to reduce brain damage in ischemic stroke.

## AUTHOR CONTRIBUTIONS

Man Cheng, Xiaoshan Liang, and Xumei Zhang designed the study and wrote the manuscript. Man Cheng and Linran Shi performed the experiments and quantification of the data. Qiang Zhang reviewed the manuscript. Liwen Zhang and Zhongying Gong analyzed the data. Suhui Luo and Xuan Wang provided intellectual contributions and participated in the discussion. All authors read and approved the final manuscript.

## CONFLICT OF INTEREST STATEMENT

The authors declare that the research was conducted in the absence of any commercial or financial relationships that could be construed as a potential conflict of interest.

## Data Availability

The data sets and/or analyzed during the current study are available from the corresponding author on request.

## References

[1] Li WA , Geng X , Ding Y . Stroke is a global epidemic: new developments in clinical and translational cerebrovascular diseases research. Neurol Res. 2017;39(6):475‐476. 28531374 10.1080/01616412.2017.1330307

[2] El Amki M , Wegener S . Improving cerebral blood flow after arterial recanalization: a novel therapeutic strategy in stroke. Int J Mol Sci. 2017;18(12):2669. 29232823 10.3390/ijms18122669 PMC5751271

[3] Ojo O , Brooke J . The use of enteral nutrition in the management of stroke. Nutrients. 2016;8(12):827. 27999383 10.3390/nu8120827 PMC5188480

[4] Garcez ML , Mina F , Bellettini‐Santos T , et al. Folic acid supplementation in the gestational phase of female rats improves age‐related memory impairment and neuroinflammation in their adult and aged offspring. J Gerontol A Biol Sci Med Sci. 2021;76(6):991‐995. 33249457 10.1093/gerona/glaa298

[5] Barichello T , Generoso JS , Simoes LR , et al. Folic acid prevented cognitive impairment in experimental pneumococcal meningitis. J Neural Transm (Vienna). 2015;122(5):643‐651. 25233798 10.1007/s00702-014-1302-3

[6] Maccioni RB , Rojo LE , Fernandez JA , Kuljis RO . The role of neuroimmunomodulation in Alzheimer's disease. Ann N Y Acad Sci. 2009;1153:240‐246. 19236346 10.1111/j.1749-6632.2008.03972.x

[7] Guest J , Bilgin A , Hokin B , Mori TA , Croft KD , Grant R . Novel relationships between B12, folate and markers of inflammation, oxidative stress and NAD(H) levels, systemically and in the CNS of a healthy human cohort. Nutr Neurosci. 2015;18(8):355‐364. 26263423 10.1179/1476830515Y.0000000041

[8] Cheng M , Yang L , Dong Z , et al. Folic acid deficiency enhanced microglial immune response via the Notch1/nuclear factor kappa B p65 pathway in hippocampus following rat brain I/R injury and BV2 cells. J Cell Mol Med. 2019;23(7):4795‐4807. 31087489 10.1111/jcmm.14368 PMC6584545

[9] Kwon HS , Koh SH . Neuroinflammation in neurodegenerative disorders: the roles of microglia and astrocytes. Transl Neurodegener. 2020;9(1):42. 33239064 10.1186/s40035-020-00221-2 PMC7689983

[10] Sofroniew MV , Vinters HV . Astrocytes: biology and pathology. Acta Neuropathol. 2010;119(1):7‐35. 20012068 10.1007/s00401-009-0619-8 PMC2799634

[11] Heneka MT , Carson MJ , El Khoury J , et al. Neuroinflammation in Alzheimer's disease. Lancet Neurol. 2015;14(4):388‐405. 25792098 10.1016/S1474-4422(15)70016-5 PMC5909703

[12] Mayo L , Trauger SA , Blain M , et al. Regulation of astrocyte activation by glycolipids drives chronic CNS inflammation. Nat Med. 2014;20(10):1147‐1156. 25216636 10.1038/nm.3681 PMC4255949

[13] Buffo A , Rolando C , Ceruti S . Astrocytes in the damaged brain: molecular and cellular insights into their reactive response and healing potential. Biochem Pharmacol. 2010;79(2):77‐89. 19765548 10.1016/j.bcp.2009.09.014

[14] Hong S , Song M‐R . STAT3 but not STAT1 is required for astrocyte differentiation. PLoS One. 2014;9(1):e86851. 24466267 10.1371/journal.pone.0086851 PMC3900679

[15] Bharadwaj U , Kasembeli MM , Robinson P , Tweardy DJ . Targeting janus kinases and signal transducer and activator of transcription 3 to treat inflammation, fibrosis, and cancer: rationale, Progress, and caution. Pharmacol Rev. 2020;72(2):486‐526. 32198236 10.1124/pr.119.018440 PMC7300325

[16] Zhao N , Xu X , Jiang Y , et al. Lipocalin‐2 may produce damaging effect after cerebral ischemia by inducing astrocytes classical activation. J Neuroinflammation. 2019;16(1):168. 31426811 10.1186/s12974-019-1556-7 PMC6699078

[17] Ahn S‐H , Heo T‐H , Jun H‐S , Choi Y . In vitro and in vivo pharmacokinetic characterization of LMT‐28 as a novel small molecular interleukin‐6 inhibitor. Asian‐Australas J Anim Sci. 2020;33(4):670‐677. 31480155 10.5713/ajas.19.0463 PMC7054612

[18] Liang N , Li S , Liang Y , et al. Clusterin inhibits Cr(VI)‐induced apoptosis via enhancing mitochondrial biogenesis through AKT‐associated STAT3 activation in L02 hepatocytes. Ecotoxicol Environ Saf. 2021;221:112447. 34175824 10.1016/j.ecoenv.2021.112447

[19] Menet CJ , Fletcher SR , Van Lommen G , et al. Triazolopyridines as selective JAK1 inhibitors: from hit identification to GLPG0634. J Med Chem. 2014;57(22):9323‐9342. 25369270 10.1021/jm501262q

[20] Sarmiento Soto M , Larkin JR , Martin C , et al. STAT3‐mediated astrocyte reactivity associated with brain metastasis contributes to neurovascular dysfunction. Cancer Res. 2020;80(24):5642‐5655. 33106335 10.1158/0008-5472.CAN-20-2251

[21] Yeung YT , Aziz F , Guerrero‐Castilla A , Arguelles S . Signaling pathways in inflammation and anti‐inflammatory therapies. Curr Pharm des. 2018;24(14):1449‐1484. 29589535 10.2174/1381612824666180327165604

[22] Chen S , Dong Z , Cheng M , et al. Homocysteine exaggerates microglia activation and neuroinflammation through microglia localized STAT3 overactivation following ischemic stroke. J Neuroinflammation. 2017;14(1):187. 28923114 10.1186/s12974-017-0963-x PMC5604224

[23] Anderson MA , Ao Y , Sofroniew MV . Heterogeneity of reactive astrocytes. Neurosci Lett. 2014;565:23‐29. 24361547 10.1016/j.neulet.2013.12.030 PMC3984948

[24] Hasel P , Rose IVL , Sadick JS , Kim RD , Liddelow SA . Neuroinflammatory astrocyte subtypes in the mouse brain. Nat Neurosci. 2021;24(10):1475‐1487. 34413515 10.1038/s41593-021-00905-6

[25] Luo P , Wang Y , Zhao C , et al. Bazedoxifene exhibits anti‐inflammation and anti‐atherosclerotic effects via inhibition of IL‐6/IL‐6R/STAT3 signaling. Eur J Pharmacol. 2021;893:173822. 33347820 10.1016/j.ejphar.2020.173822

[26] Endres M , Ahmadi M , Kruman I , Biniszkiewicz D , Meisel A , Gertz K . Folate deficiency increases postischemic brain injury. Stroke. 2005;36(2):321‐325. 15625295 10.1161/01.STR.0000153008.60517.ab

[27] Patabendige A , Singh A , Jenkins S , Sen J , Chen R . Astrocyte activation in neurovascular damage and repair following ischaemic stroke. Int J Mol Sci. 2021;22(8):4280. 33924191 10.3390/ijms22084280 PMC8074612

[28] Novochadlo M , Goldim MP , Bonfante S , et al. Folic acid alleviates the blood brain barrier permeability and oxidative stress and prevents cognitive decline in sepsis‐surviving rats. Microvasc Res. 2021;137:104193. 34062190 10.1016/j.mvr.2021.104193

[29] Gu Y , He M , Zhou X , et al. Endogenous IL‐6 of mesenchymal stem cell improves behavioral outcome of hypoxic‐ischemic brain damage neonatal rats by suppressing apoptosis in astrocyte. Sci Rep. 2016;6:18587. 26766745 10.1038/srep18587 PMC4725911

[30] Yang X , Yv Q , Ye F , et al. Echinacoside protects dopaminergic neurons through regulating IL‐6/JAK2/STAT3 pathway in Parkinson's disease model. Front Pharmacol. 2022;13:848813. 35281889 10.3389/fphar.2022.848813 PMC8914071

[31] Yao D , Zhou Z , Wang P , et al. MiR‐125‐5p/IL‐6R axis regulates macrophage inflammatory response and intestinal epithelial cell apoptosis in ulcerative colitis through JAK1/STAT3 and NF‐kappaB pathway. Cell Cycle. 2021;20(23):2547‐2564. 34747340 10.1080/15384101.2021.1995128 PMC8794517

[32] Quintás‐Cardama A , Vaddi K , Liu P , et al. Preclinical characterization of the selective JAK1/2 inhibitor INCB018424: therapeutic implications for the treatment of myeloproliferative neoplasms. Blood. 2010;115(15):3109‐3117. 20130243 10.1182/blood-2009-04-214957 PMC3953826

[33] Vainchenker W , Dusa A , Constantinescu SN . JAKs in pathology: role of Janus kinases in hematopoietic malignancies and immunodeficiencies. Semin Cell Dev Biol. 2008;19(4):385‐393. 18682296 10.1016/j.semcdb.2008.07.002

[34] Zhong Z , Wen Z , Darnell JE Jr . Stat3: a STAT family member activated by tyrosine phosphorylation in response to epidermal growth factor and interleukin‐6. Science. 1994;264(5155):95‐98. 8140422 10.1126/science.8140422

[35] Campbell IL . Cytokine‐mediated inflammation, tumorigenesis, and disease‐associated JAK/STAT/SOCS signaling circuits in the CNS. Brain Res Brain Res Rev. 2005;48(2):166‐177. 15850655 10.1016/j.brainresrev.2004.12.006

[36] Brenner M , Messing A . Regulation of GFAP expression. ASN Neuro. 2021;13:81206. 10.1177/1759091420981206 PMC7897836 33601918

[37] Ralay Ranaivo H , Patel F , Wainwright MS . Albumin activates the canonical TGF receptor‐smad signaling pathway but this is not required for activation of astrocytes. Exp Neurol. 2010;226(2):310‐319. 20854815 10.1016/j.expneurol.2010.09.005

[38] Jiao M , Yin K , Zhang T , et al. Effect of the SSeCKS‐TRAF6 interaction on gastrodin‐mediated protection against 2,3,7,8‐tetrachlorodibenzo‐p‐dioxin‐induced astrocyte activation and neuronal death. Chemosphere. 2019;226:678‐686. 30959452 10.1016/j.chemosphere.2019.04.003

[39] Ceyzeriat K , Abjean L , Carrillo‐de Sauvage MA , Ben Haim L , Escartin C . The complex STATes of astrocyte reactivity: how are they controlled by the JAK‐STAT3 pathway? Neuroscience. 2016;330:205‐218. 27241943 10.1016/j.neuroscience.2016.05.043

[40] Kang W , Balordi F , Su N , Chen L , Fishell G , Hebert JM . Astrocyte activation is suppressed in both normal and injured brain by FGF signaling. Proc Natl Acad Sci U S A. 2014;111(29):E2987‐E2995. 25002516 10.1073/pnas.1320401111 PMC4115557

[41] Takizawa T , Nakashima K , Namihira M , et al. DNA methylation is a critical cell‐intrinsic determinant of astrocyte differentiation in the fetal brain. Dev Cell. 2001;1(6):749‐758. 11740937 10.1016/s1534-5807(01)00101-0

[42] Brenner M , Messing A , Olsen ML . AP‐1 and the injury response of the GFAP gene. J Neurosci Res. 2019;97(2):149‐161. 30345544 10.1002/jnr.24338 PMC6289842

[43] Crider KS , Yang TP , Berry RJ , Bailey LB . Folate and DNA methylation: a review of molecular mechanisms and the evidence for folate's role. Adv Nutr. 2012;3(1):21‐38. 22332098 10.3945/an.111.000992 PMC3262611

[44] Monsour M , Borlongan CV . The central role of peripheral inflammation in ischemic stroke. J Cereb Blood Flow Metab. 2023. doi:10.1177/0271678X221149509 PMC10108194 36601776

[45] Zhou SY , Guo ZN , Zhang DH , Qu Y , Jin H . The role of pericytes in ischemic stroke: Fom cellular functions to therapeutic targets. Front Mol Neurosci. 2022;15:866700. 35493333 10.3389/fnmol.2022.866700 PMC9043812

[46] Liu LR , Liu JC , Bao JS , Bai QQ , Wang GQ . Interaction of microglia and astrocytes in the neurovascular unit. Front Immunol. 2020;11:1024. 32733433 10.3389/fimmu.2020.01024 PMC7362712

